# CHLD score, a new score based on traditional risk factor evaluation and long-term cardiovascular outcomes in patients with systemic sclerosis

**DOI:** 10.1038/s41598-021-99215-x

**Published:** 2021-10-01

**Authors:** Klaudia Gieszczyk-Strózik, Maciej T. Wybraniec, Małgorzata Widuchowska, Ligia Brzezińska-Wcisło, Przemysław Kotyla, Eugeniusz Kucharz, Katarzyna Mizia-Stec

**Affiliations:** 1 First Department of Cardiology, School of Medicine in Katowice, Medical University of Silesia, 47 Ziołowa St., 40-635 Katowice, Poland; 2 Upper Silesia Medical Centre, Katowice, Poland; 3 Department of Internal Medicine, Rheumatology and Clinical Immunology; School of Medicine in Katowice, Medical University of Silesia, Katowice, Poland; 4 Department of Dermatology, School of Medicine in Katowice, Medical University of Silesia, Katowice, Poland

**Keywords:** Cardiomyopathies, Heart failure

## Abstract

The aim of the study was to assess the predictors of major adverse cardiovascular events (MACE) in patients with systemic sclerosis (SSc) without pulmonary arterial hypertension. The study comprised 68 patients with SSc who were followed up for the median time of 99 (96; 107) months. The main exclusion criteria involved tricuspid regurgitation maximal velocity > 2.8 m/s and structural heart disease. At baseline the patients underwent clinical assessment of cardiovascular risk factors, 6-min walk test, transthoracic echocardiography and biomarker testing, including growth differentiation factor 15 (GDF-15). The primary composite endpoint was onset of MACE defined as death, myocardial infarction, myocardial revascularization and hospitalization for heart failure. The follow-up consisted of outpatient visits at 1 year intervals and telephone interview every 6 months. The baseline analysis revealed that chronic kidney disease (HR 28.13, 95%CI 4.84–163.38), lung fibrosis on high resolution computed tomography (HR 4.36, 95%CI 1.04–18.26) and GDF-15 concentration (unit HR 1.0006, 95%CI 1.0002–1.0010) were independent predictors of MACE occurrence. CHLD (Chronic kidney disease, Hypertension, hyperLipidaemia, Diabetes mellitus) score was formulated which assigned 1 point for the presence of arterial hypertension, hyperlipidaemia, diabetes mellitus and chronic kidney disease. After inclusion of CHLD score in Cox proportional model, it remained the only independent predictor of MACE onset (unit HR per 1 point 3.46; 95%CI 2.06–5.82, p < 0.0001). Joint assessment of traditional risk factors in the form of CHLD score may serve as a reliable predictor of long-term outcome in patients with SSc without pulmonary arterial hypertension.

## Introduction

Systemic sclerosis (SSc) is a multiorgan disease with significant morbidity that is characterised by fibrosis of the internal organs and skin, autoimmune phenomena, and vasculopathy, significantly affecting cardiovascular system and long-term outcome^1–3^. While the vasculopathy in the course of SSc is usually associated with the development of pulmonary arterial hypertension, the evidence concerning its association with acute coronary syndromes and heart failure has accumulated recently^4–7^. It has been demonstrated that cardiovascular events account for one-fifth causes of death among SSc patients^8^. The data on long-term follow-up revealed that patients with SSc are prone to twofold higher risk of adverse cardiovascular events than patients without SSc in a matched population^4^. Also, acute myocardial infarction (AMI) in patients with SSc was shown to be associated with higher risk of recurrent adverse cardiovascular events and death, but not bleeding in comparison to other patients with AMI^9^. Of note, patients with SSc and adverse cardiovascular events had much worse prognosis than patients with other systemic connective tissue diseases, such as rheumatoid arthritis or systemic lupus erythematosus^10^ The cardiac involvement in SSc is thought to be related to microvascular dysfunction and resultant subclinical systolic dysfunction reflected by impaired left ventricular (LV) global longitudinal strain, which even precede development of pulmonary hypertension^11^. Patients with cardiovascular incidents were characterized by cardiac magnetic resonance alterations, such as early and late gadolinium enhancement^12^. Given this evidence of subclinical cardiac involvement, the present study aimed to evaluate the predictors of major adverse cardiovascular events (MACE) in patients with SSc without pulmonary arterial hypertension, with a special focus on traditional cardiovascular risk factors.

## Materials and methods

The article represents an observational study comprising 68 patients with confirmed SSc meeting American College of Rheumatology/European League Against Rheumatism (ACR-EULAR) classification criteria^13^ with excluded pulmonary hypertension, who were meticulously assessed and followed up for the median time of 97 (74.5; 105) months.

The exclusion criteria comprised (a) tricuspid regurgitation maximal velocity > 2.8 m/s; (b) age < 18 and > 75 years; (c) established coronary artery disease at inclusion; (d) history of acute coronary syndrome and percutaneous coronary intervention; (e) history of transient ischemic attack or stroke; (f) peripheral artery disease; (g) heart failure with reduced and mid-range ejection fraction (HFrEF or HFmrEF); (h) severe valvular heart disease; (i) chronic dialysis therapy; (j) pregnancy; (k) active neoplastic disease.

Diagnostic workup involved meticulous review of past medical history, symptoms, physical examination, 6-min walk test (6MWT), transthoracic echocardiography with tissue Doppler imaging, measurement of C-reactive protein, fetuin-A, growth differentiation factor 15 (GDF-15) and N-terminal pro-B-type natriuretic peptide (NT-proBNP). All the patients underwent 24-h 3-lead electrocardiographic monitoring, nitrate- (NMD) and flow-mediated dilation (FMD) and photo-plethysmography so as to establish brachial-radial pulse wave velocity (PWV). The precise description of diagnostic methods can be found in formerly published study from our institution^14^. Chronic kidney disease (CKD) was defined as presence of estimated glomerular filtration rate (eGFR) < 60 ml/min/1.73 m^2^. Arterial hypertension was defined as history of arterial hypertension, confirmed blood pressure > 140/90 mmHg on two separate days during index hospitalization or antihypertensive treatment.

The primary composite endpoint was onset of MACE defined as all-cause death, myocardial infarction, myocardial revascularization and hospitalization for heart failure. The secondary endpoint was all-cause death in the studied population.

The follow-up was properly structured and involved screening visits at 1 year intervals and telephone interview every 6 months.

The study adhered to Declaration of Helsinki Guidelines and was accepted by the Ethics Committee of Medical University of Silesia in Katowice. All study participants gave written informed consent to participation in the study.

Statistical analysis was performed using SPSS v.25.0 software (IBM Corp. Armonk. NY) and MedCalc v.14.8.1 software (MedCalc Software, Ostend, Belgium). In case of normal distribution, student’s t test or analysis of variance (ANOVA) tests were applied, while in non-normally distributed variables two-tailed Mann–Whitney U or Kruskal–Wallis tests were utilized. The significance of proportions in contingency tables were calculated using Chi-square test with Bonferroni adjustment. All the variables with p < 0.1 in univariable model were incorporated into cox proportional hazards model, except for the data on pharmacotherapy (valid only for the moment of inclusion) and all the colinear variables (e.g. components of the CHLD score) in order to establish independent predictors of MACE and all-cause death. The cox proportional hazards model used stepwise approach. The final model comprised CHLD score, body mass index, age, high resolution computed tomography (HRCT) lung fibrosis, GDF-15 concentration, forced vital capacity and total lung capacity. Kaplan–Meier survival curves with log-rank test were plotted for significant variables in univariable analysis.

## Results

The analysis covered 68 patients with systemic sclerosis with mean age of 52.3 ± 11.6 years and predominance of females (n = 55; 80.9%). The study population was characterized by pronounced cardiovascular risk factors with high prevalence of arterial hypertension (n = 47, 69%), hyperlipidaemia (n = 42, 62%), diabetes mellitus (n = 12, 18%), and chronic kidney disease (n = 20, 29.4%). Obesity was present in 5 patients (7.4%). The localized subset of SSc was diagnosed in 33 patients (49%) and diffuse subset in 35 participants (52%).

During the median follow-up time of 99 (96; 107) months, MACE was diagnosed in 32 patients (47%). Ten patients died (15%), nine patients (13%) developed myocardial infarction, 20 patients (29%) underwent percutaneous coronary intervention, 4 patients (6%) underwent pacemaker implantation, 16 patients (24%) experienced heart failure symptoms.

The comparison of different demographic and clinical variables between MACE and non-MACE group was highlighted in Table 1. The analysis revealed that classic cardiovascular risk factors like arterial hypertension, were more frequent in patients who exhibited MACE (Table 1). Kaplan–Meier survival curves have shown that arterial hypertension (log-rank p = 0.0001), hyperlipidaemia (p = 0.0025), diabetes mellitus (p = 0.0036) and chronic kidney disease (p < 0.0001) stratified patients in terms of MACE occurrence. The results of univariable analysis are presented in Table 2.

**Table 1:** Demographic and clinical characteristics in patients with and without major adverse cardiovascular events in long-term follow-up.

	No MACE N = 36	MACE N = 32	P-value
	Mean ± SD or median (1Q-3Q) or n (%)	Mean ± SD or median (1Q-3Q) or n (%)	
Male sex	4 (11%)	9 (28%)	0.075^a^
Obesity	1 (3%)	4 (13%)	0.125^a^
Arterial hypertension	17 (47%)	30 (94%)	< 0.001^a^
Diabetes mellitus type 2	3 (8%)	9 (28%)	0.033^a^
Hiperlipidaemia	15 (42%)	27 (84%)	< 0.001^a^
Chronic kidney disease	2 (6%)	18 (56%)	< 0.001^a^
LV wall motion abnormalities	1 (3%)	2 (7%)	0.450^a^
**SSc subset**			
Limited	16 (44%)	17 (53%)	0.475^a^
Diffuse	20 (56%)	15 (47%)	
Autologous stem cell transplantation	1 (3%)	4 (13%)	0.125^a^
Modified Rodnan Score	6.00 (4.50; 10.00)	8.00 (5.00; 11.00)	0.688^b^
Proton pump inhibitor	17 (47%)	19 (59%)	0.316^a^
NSAIDs	0 (0%)	1 (3%)	0.285^a^
Diuretics	3 (9%)	10 (31%)	0.019^a^
Calcium channel blockers	8 (22%)	15 (47%)	0.032^a^
ACE-inhibitors	10 (28%)	16 (50%)	0.060^a^
Angiotensin receptor blockers	2 (6%)	1 (3%)	0.626^a^
Beta blocker	5 (14%)	3 (9%)	0.536^a^
ANA	33 (97%)	27 (87%)	0.132^a^
ACA	6 (19%)	3 (10%)	0.329^a^
Scl70	17 (52%)	17 (59%)	0.575^a^
HRCT lung fibrosis	8 (27%)	14 (50%)	0.067^a^
HRCT ground glass opacification	6 (20%)	7 (25%)	0.648^a^
**Capillaroscopy pattern**			
Early	5 (17%)	3 (13%)	0.815^a^
Active	9 (30%)	9 (38%)	
Late	16 (53%)	12 (50%)	
Age [years]	47.43 ± 11.33	57.34 ± 9.96	0.001^b^
Body mass index [kg/m^2^]	23.15 ± 2.99	25.12 ± 3.84	0.037^b^
Waist-hip ratio	0.80 (0.78; 0.86)	0.86 (0.77; 0.93)	0.100^b^
CHLD score	0 (0; 1)	2 (1; 2.5)	< 0.001^b^
C-reactive protein [ng/ml]	1668.00 (738.84; 2745.80)	3584.10 (1198.70; 7632.00)	0.084^b^
Fetuin-A [µg/ml]	370.37 ± 116.44	347.24 ± 111.70	0.542^b^
GDF-15 [pg/ml]	2023.60 (1463.00; 3294.50)	3210.20 (2087.20; 4513.10)	0.027^b^
NT-proBNP [pg/ml]	778.19 (429.33; 2037.46)	1335.50 (590.99; 2675.61)	0.150^b^
PWV (m/s)	8.20 ± 1.68	8.23 ± 1.45	0.770^b^
FMD [%]	15.73 (9.73; 22.44)	15.49 (13.21; 18.68)	0.940^b^
NMD [%]	28.88 (23.61; 37.36)	25.32 (19.92; 31.21)	0.150^b^
6MWT [m]	475 (420; 505)	422.50 (400; 475)	0.018^b^
DLCO (% predicted)	86.92 ± 13.00	59.33 ± 22.22	0.018^b^
FVC (% predicted)	100.64 ± 6.12	80.75 ± 12.07	0.001^b^
FEV1 (% predicted)	103.56 ± 5.10	87.33 ± 14.11	0.033^b^
TLC (% predicted)	98.11 ± 16.43	73.86 ± 12.13	0.003^b^
LAd [mm]	30.67 ± 3.99	33.62 ± 4.22	0.005^b^
LVEF [%]	61.72 ± 5.21	60.16 ± 6.16	0.247^b^
RAA	13.24 ± 3.42	14.92 ± 4.02	0.092^b^
LAA	15.18 ± 3.20	16.80 ± 3.39	0.052^b^
E′ mean	0.13 (0.09; 0.16)	0.10 (0.08; 0.13)	0.040^b^
E/A	1.32 (1.00; 1.60)	0.81 (0.73; 1.00)	< 0.001^b^
E′ ivs < 7	28 (78%)	27 (84%)	0.490^a^
E′ lat < 10	10 (28%)	16 (50%)	0.060^a^
E/e′	6.21 (5.31; 8.24)	6.86 (4.90; 9.69)	0.530^b^
E/e′ > 14	1 (3%)	2 (6%)	0.486^a^
RVSP	32.82 ± 7.31	34.92 ± 9.89	0.406^b^
CHLD score	0 (0; 1)	2 (1.0; 2.50)	< 0.001^b^
Follow-up time [months]	99.5 (96.0; 108.5)	98 (85.5; 106.0)	0.191^b^

6MWT: 6-min walk test; ACE: angiotensin-converting enzyme; ANA: antinuclear antibodies; ACA: anticentromere antibodies DLCO: diffusing capacity for carbon monoxide; FVC: forced vital capacity; GDF-15: growth differentiation factor 15; HRCT: high-resolution chest tomography; NSAIDs: non-steroidal anti-inflammatory drugs; NT-proBNP: N-terminal pro-B-type natriuretic peptide; FMD: flow-mediated dilation; NMD: nitrate-mediated dilation; PWV: pulse wave velocity; CHLD: chronic kidney disease, arterial hypertension, hyperlipidaemia and diabetes mellitus score; LA-left atrial diameter; LAA: left atrial area; LVEF: left ventricular ejection fraction; LV: left ventricular; RAA: right atrial area; RVSP: right ventricular systolic pressure; SSc: systemic sclerosis; TLC: total lung capacity.

^a^Chi-square test; ^b^Mann-Whitney U test.

**Table 2:** Univariable and multivariable cox proportional hazards analysis.

Variable	Univariable			Multivariable		
	OR	95%CI	P	OR	95%CI	P
Male sex	1.42	0.66–3.08	0.38			
Obesity	2.62	0.90–7.56	0.08			
Arterial hypertension	11.93	2.84–50.15	0.001			
Diabetes mellitus type 2	2.99	1.38–6.53	0.006			
Hiperlipidaemia	3.88	1.50–10.03	0.006			
Chronic kidney disease	10.75	4.66–24.80	< 0.0001			
LV wall motion abnormalities	2.46	0.58–10.41	0.23			
Diffuse SSc	0.79	0.39–1.58	0.51			
Autologous stem cell transplantation	1.68	0.59–4.78	0.34			
Modified Rodnan Score	1.003	0.97–1.04	0.88			
Proton pump inhibitor	1.22	0.61–2.47	0.57			
NSAIDs	9.06	1.13–72.89	0.04			
Diuretics	2.22	1.05–4.70	0.04			
Calcium channel blockers	2.28	1.14–4.58	0.02			
ACE-inhibitors	1.92	0.96–3.83	0.07			
Angiotensin receptor blockers	0.65	0.09–4.73	0.67			
Beta blocker	0.82	0.25–2.67	0.74			
ANA	0.48	0.17–1.37	0.17			
ACA	0.58	0.18–1.91	0.37			
Scl70	1.20	0.58–2.51	0.63			
HRCT lung fibrosis	1.76	0.84–3.70	0.14			
HRCT ground glass opacification	1.22	0.52–2.86	0.65			
Age [per 1 year]	1.08	1.04–1.12	0.0001			
Body mass index [per 1 kg/m^2^]	1.13	1.02–1.25	0.02			
Waist-hip ratio	0.96	0.76–1.20	0.70			
CHLD score [per 1 pt]	2.93	2.02–4.26	< 0.0001	3.46	2.06–5.82	< 0.0001
C-reactive protein [per 1 ng/ml]	1.00	1.00–1.00	0.24			
Fetuin-A [per 1 µg/ml]	1.00	0.99–1.00	0.80			
GDF-15 [per 1 pg/ml]	1.00	1.00–1.00	0.03			
NT-proBNP [per 1 pg/ml]	1.00	0.99–1.00	0.20			
PWV (per 1 m/s)	1.04	0.82–1.32	0.76			
FMD [per 1%]	1.01	0.97–1.05	0.61			
NMD [per 1%]	0.98	0.96–1.01	0.22			
6MWT [per 1 m]	0.99	0.99–1.00	0.14			
DLCO [per 1% predicted]	0.96	0.92–0.99	0.03			
FVC [per 1% predicted]	0.95	0.91–0.99	0.02			
FEV1 [per 1% predicted]	0.95	0.90–1.01	0.09			
TLC [per 1% predicted]	0.95	0.89–0.99	0.049			

6MWT: 6-min walk test; ACE: angiotensin-converting enzyme; ANA: antinuclear antibodies; ACA: anticentromere antibodies; DLCO: diffusing capacity for carbon monoxide; FVC: forced vital capacity; GDF-15: growth differentiation factor 15; HRCT: high-resolution chest tomography; NSAIDs: non-steroidal anti-inflammatory drugs; NT-proBNP: N-terminal pro-B-type natriuretic peptide; FMD: flow-mediated dilation; NMD: nitrate-mediated dilation; PWV: pulse wave velocity; CHLD: chronic kidney disease, arterial hypertension, hyperlipidaemia and diabetes mellitus score; LA-left atrial diameter; LAA: left atrial area; LVEF: left ventricular ejection fraction; LV: left ventricular RAA: right atrial area; RVSP: right ventricular systolic pressure; SSc: systemic sclerosis; TLC: total lung capacity.

**Figure 1:**
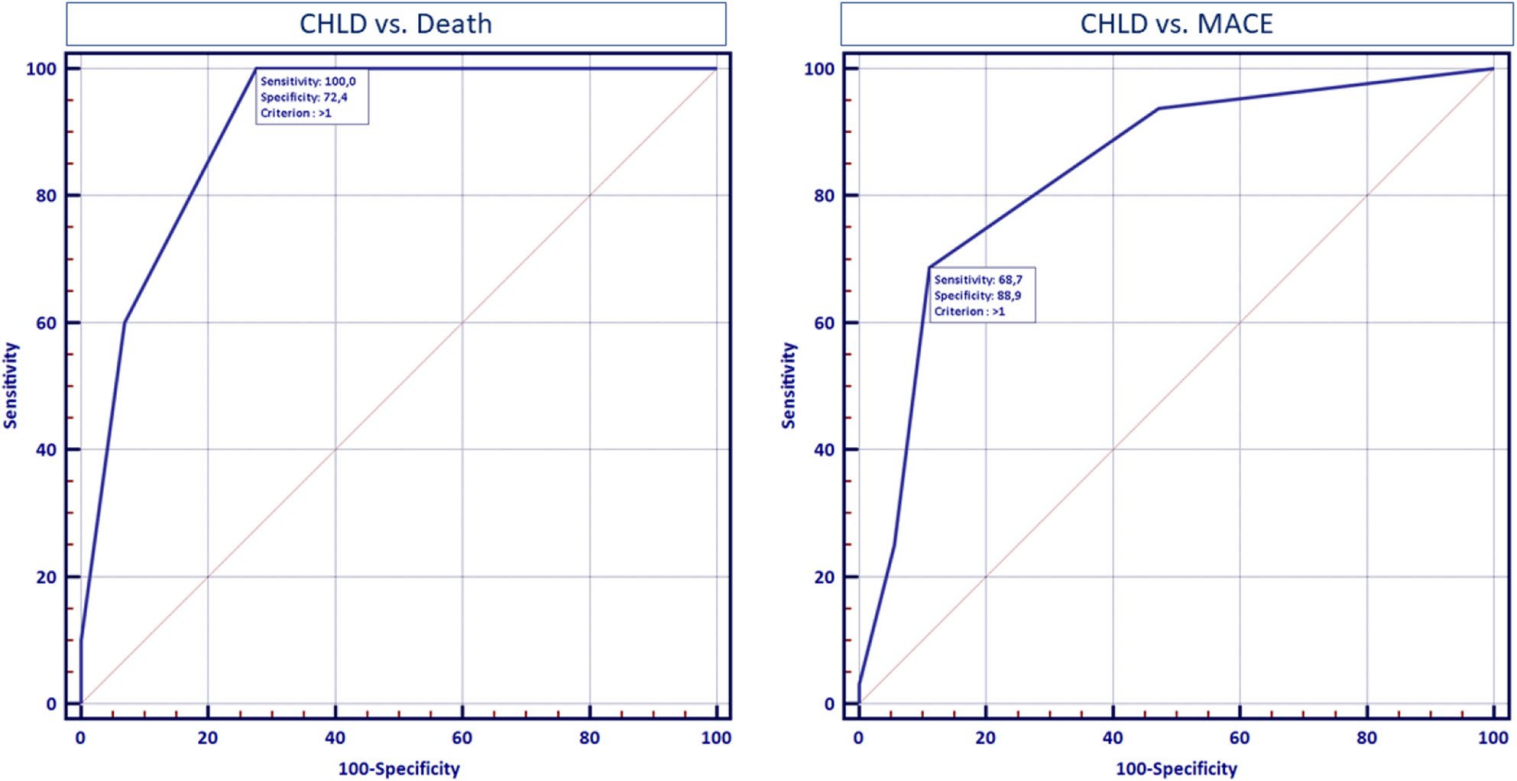
Receiver operating characteristics curve of application of CHLD score for prediction of death and major adverse cardiovascular events. CHLD score: C-chronic kidney disease-1 pt; H: arterial hypertension-1 pt; L: hyperLipidemia-1 pt; D: diabetes mellitus-1 pt; MACE: major adverse cardiovascular events.

The initial Cox proportional hazards model revealed that CKD (HR 28.13, 95%CI 4.84–163.38, p = 0.0002), HRCT lung fibrosis (HR 4.36, 95%CI 1.04–18.26, p = 0.045) and GDF-15 concentration (unit HR 1.0006, 95%CI 1.0002–1.0010, p = 0.003) were independent predictors of MACE occurrence. Subsequently, we have constructed a CHLD score which assigned 1 point for the presence of arterial hypertension, hyperlipidaemia, diabetes mellitus and CKD. After inclusion of CHLD score in Cox proportional model, it remained the only independent predictor of MACE onset (Table 2; unit HR per 1 point 3.46; 95%CI 2.06–5.82, p < 0.0001). Receiver operating curve (ROC) analysis (Fig. 1) showed that CHLD score accurately predicted MACE (AUC 0.839; 95%CI 0.745–0.932, p < 0.0001).

The univariable analysis of secondary endpoint revealed that death was accurately predicted by diabetes mellitus (OR = 5.75, 95%CI 1.66–19.96, p = 0.0061), use of calcium channel blockers (OR = 5.09, 95%CI 1.32–19.55, p = 0.0184), lung fibrosis on HRCT (OR = 5.43, 95%CI 1.10–26.71, p = 0.04), age (per 1 year OR = 1.13, 95%CI 1.06–1.22, p = 0.0004), CHLD score (per 1 point OR = 4.68, 95%CI 2.28–9.62, p < 0.0001). Cox proportional hazards model revealed that CHLD score was independently associated with all-cause death during study follow-up (per 1 point OR = 20.02, 95%CI 3.39–118.26, p = 0.001). The ROC curve analysis showed that CHLD score accurately predicted all-cause death (AUC 0.914, 95%CI 0.85–0.98, p < 0.0001) on follow-up.

## Discussion

The above-mentioned results underscore the importance of traditional cardiovascular risk factors, along with acknowledged disease-specific determinants of long-term prognosis such as lung fibrosis. The results of our study show similar mortality rate to hitherto reports, namely study by Poormoghim et al., who showed 18% mortality rate in a 10-year follow-up^4^. In contrast, average risk of 10-year cardiovascular death without risk factors in broad Polish population of females based on SCORE algorithm is roughly 1%^15^. Still, in line with our data, advance pulmonary fibrosis and age > 50 years, apart from tendon friction rub and arthritis, constituted independent predictors of long-term outcome, yet the study did not underscore the importance of classic cardiovascular risk factors^4^. The combined cardiovascular risk in the form of CHLD score in our analysis accurately predicted both composite endpoint of MACE and mortality alone (Fig. 1).

The case–control study by Kurmann and coworkers on 78 patients with SSc and 156 sex- and age-matched subjects with a follow-up time comparable to our report and demonstrated that SSc subjects had a significantly higher risk of myocardial infarction (5.9% vs. 3.9%, HR 4.88, 95%CI 1.21–19.72) and congestive heart failure (12.8% vs 7.2%, HR 3.6, 95%CI 1.34–9.71)^8^, although the determinants of the endpoint were not evaluated.

It is worth to emphasize that patients with SSc have higher rate of acute coronary syndromes, but also their treatment is fraught with higher risk of complications. SSc patients undergoing percutaneous coronary interventions were more likely to exhibit in-hospital complications than patients without connective tissue disorders, with much more higher in-hospital all-cause mortality (OR 1.32, 95%CI 1.03–1.71)^16^.

Our results contribute to the understanding of the role of basic cardiovascular risk factors that should be jointly evaluated in every individual, not only patients with SSc. CHLD represents any easy predictive tool that may help assess the cardiovascular risk of patients with SSc.

It is, however, important to interpret the results of the study with due caution on account of several limitations. First, the study population was relatively low count, which might have had impact on the results of the Cox proportional hazards model. Second, the composite endpoint of MACE did not cover stroke. Third, MACE included all-cause death and it was responsible for roughly 1/3 of all events during the follow-up.

The results of the present study highlight the need for meticulous cardiovascular supervision of patients with SSc, extending further than just the routine screening for pulmonary arterial hypertension. CHLD score represents an independent predictor of long-term outcome in patients with SSc in the study population.
